# Role of the fission yeast cell integrity MAPK pathway in response to glucose limitation

**DOI:** 10.1186/1471-2180-13-34

**Published:** 2013-02-11

**Authors:** Marisa Madrid, Jesús Fernández-Zapata, Laura Sánchez-Mir, Teresa Soto, Alejandro Franco, Jero Vicente-Soler, Mariano Gacto, José Cansado

**Affiliations:** 1 Yeast Physiology Group, Department of Genetics and Microbiology, Facultad de Biología, Universidad de Murcia, 30071 Murcia, Spain

**Keywords:** Fission yeast, Glucose, MAPK, Pmk1, Sty1

## Abstract

**Background:**

Glucose is a signaling molecule which regulates multiple events in eukaryotic organisms and the most preferred carbon source in the fission yeast *Schizosaccharomyces pombe*. The ability of this yeast to grow in the absence of glucose becomes strongly limited due to lack of enzymes of the glyoxylate cycle that support diauxic growth. The stress-activated protein kinase (SAPK) pathway and its effectors, Sty1 MAPK and transcription factor Atf1, play a critical role in the adaptation of fission yeast to grow on alternative non-fermentable carbon sources by inducing the expression of *fbp1*^*+*^ gene, coding for the gluconeogenic enzyme fructose-1,6-bisphosphatase. The cell integrity Pmk1 pathway is another MAPK cascade that regulates various processes in fission yeast, including cell wall construction, cytokinesis, and ionic homeostasis. Pmk1 pathway also becomes strongly activated in response to glucose deprivation but its role during glucose exhaustion and ensuing adaptation to respiratory metabolism is currently unknown.

**Results:**

We found that Pmk1 activation in the absence of glucose takes place only after complete depletion of this carbon source and that such activation is not related to an endogenous oxidative stress. Notably, Pmk1 MAPK activation relies on *de novo* protein synthesis, is independent on known upstream activators of the pathway like Rho2 GTPase, and involves PKC ortholog Pck2. Also, the Glucose/cAMP pathway is required operative for full activation of the Pmk1 signaling cascade. Mutants lacking Pmk1 displayed a partial growth defect in respiratory media which was not observed in the presence of glucose. This phenotype was accompanied by a decreased and delayed expression of transcription factor Atf1 and target genes *fbp1*^+^ and *pyp2*^+^. Intriguingly, the kinetics of Sty1 activation in Pmk1-less cells was clearly altered during growth adaptation to non-fermentable carbon sources.

**Conclusions:**

Unknown upstream elements mediate Pck2-dependent signal transduction of glucose withdrawal to the cell integrity MAPK pathway. This signaling cascade reinforces the adaptive response of fission yeast to such nutritional stress by enhancing the activity of the SAPK pathway.

## Background

In addition to its role as energy source, glucose is a powerful signaling molecule which modulates many cellular responses in eukaryotic organisms, ranging from cell cycle control and differentiation to transcriptional and translational regulation [1]. The regulatory pathways involved in such signaling become particularly patent in simple eukaryotic organisms like budding or fission yeasts, where this sugar is the preferred carbon source for vegetative growth [2]. In the fission yeast *Schizosaccharomyces pombe* glucose may be fermented under aerobic conditions (Crabtree effect), and a reduction in its concentration strongly affects cell metabolism and gene expression [3]. Moreover, fission yeast cells lack enzymes of the glyoxylate cycle to maintain diauxic growth in the absence of glucose, and this feature limits to glycerol or gluconate their ability to grow on non-sugar carbon sources [4,5]. Hence, as soon as glucose disappears and respiration of the fermentation products becomes impaired *S*. *pombe* undergoes a nutritional stress [3].

Evidence has accumulated to support a key role of mitogen-activated protein kinase (MAPK) signaling pathways in the response of eukaryotic cells against environmental alterations and stress conditions [6]. In particular, the stress-activated protein kinase (SAPK) pathway, which is one of the three MAPK cascades present in fission yeast, plays a critical function during the modulation of the general cellular response to stress. The central element of this pathway is MAPK Sty1, ortholog to other SAPK members in mammalian cells like p38 and JNK, which results activated in response to multiple stressful conditions [7,8]. A main target of the SAPK pathway is transcription factor Atf1, a protein containing a leucine zipper domain (bZIP) and homologue to transcriptional factor ATF-2 of higher cells, which associates *in vivo* to, and is phosphorylated by Sty1 during stress [9]. Activated Atf1 induces the expression of a group of genes forming part of the Core Environmental Stress Response (CESR), whose products participate in the adaptive cell response [10]. Glucose starvation is an environmental stress able to activate the SAPK pathway in *S*. *pombe*[11,12], and mutants lacking either Sty1 or Atf1 are unable to grow on alternative non-fermentable carbon sources due to failure to induce the *fbp1*^+^ gene, coding for the gluconeogenic enzyme fructose-1,6-bisphosphatase [13]. Expression of this gene becomes strongly induced by activated Atf1 in the absence of glucose, whereas high glucose concentrations promote increased intracellular cAMP levels and full repression of *fbp1*^+^ due to the activity Pka1, the catalytic subunit of protein kinase A [13]. Pka1 phosphorylates and negatively regulates the activity of Rst2, a transcription factor which, together with Atf1, is responsible for the induced expression of *fbp1*^+^ when glucose is missing [14].

The cell integrity pathway is another MAPK cascade that in *S*. *pombe* regulates processes like cell wall construction and maintenance during stress, vacuole fusion, cytokinesis, morphogenesis, and ionic homeostasis [8,15,16]. Pmk1, the effector MAPK of this signaling module which also includes Mkh1 (MAPKKK) and Pek1/Skh1 (MAPKK), is ortholog to human ERK1/2, and becomes activated in response to a variety of adverse osmotic conditions, cell wall damage, oxidative stress, and glucose withdrawal [17,18]. Rho2, one of the six Rho GTPases found in fission yeast proteome (Rho1 to Rho5, and Cdc42), is a main positive upstream regulator of the cell integrity pathway whose activity is mediated through Pck2, one of the two orthologs of protein kinase C (PKC) present in this organism [18,19]. However, although Rho2 and Pck2 are the only known upstream activators of Pmk1, the existence of Pmk1 activity in the absence of both components indicates that the MAPK cascade is branched, with other elements acting upstream this pathway [18]. Some studies have suggested that the essential GTPase Rho1 might also modulate the activity Pmk1 by acting upstream of Pck2 [20]. The fact that both Sty1 and Pmk1 are activated in response to similar stimuli suggests the existence of cross-talk between both signaling cascades. In this context, we have shown that MAPK phosphatases Pyp1, Pyp2, and Ptc1 and Ptc3, whose transcriptional induction is dependent on Sty1-Atf1 function, associate *in vivo* and dephosphorylate activated Pmk1 [21]. Also, Atf1, which is the main target of Sty1, is phosphorylated by Pmk1 under cell wall damage, although the number of identified genes whose expression is induced in a Pmk1-Atf1-dependent fashion appears to be scarce [8,22]. In this work we investigated the role of the cell integrity pathway during glucose exhaustion in fission yeast. The results suggest that a specific mechanism regulates MAPK function during this particular stress and unveil the existence of a new crosstalk mechanism whereby activated Pmk1 reinforces growth adaptation to alternative carbon sources by enhancing the activity of the SAPK pathway.

## Results

### Pmk1 activation in response to glucose deprivation

**Figure 1:**
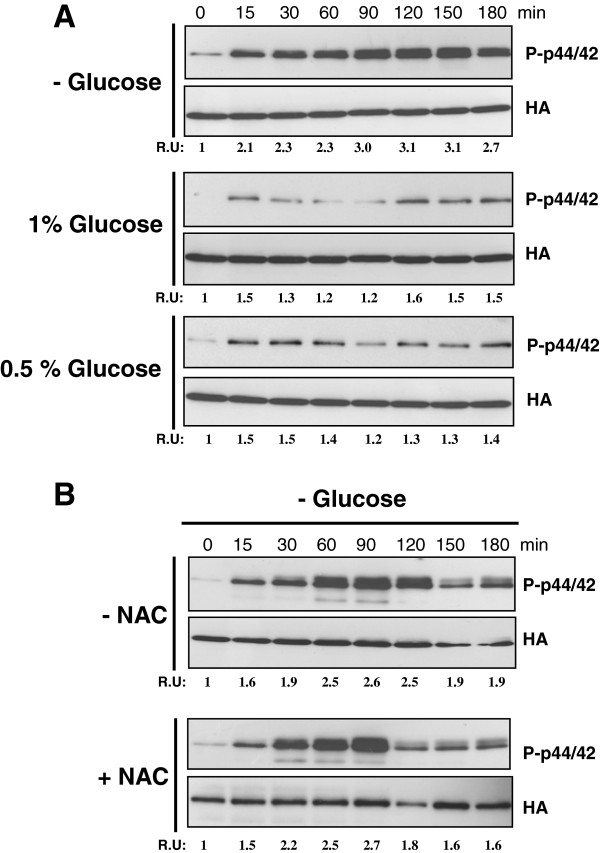
**Activation of the Pmk1 pathway in response to glucose deprivation. A**. Strain MI200 (Pmk1-Ha6H) was grown in YES medium plus 7% glucose to early-log phase and transferred to the same medium with 3% glycerol (upper panel), 2.5% glycerol plus 1% glucose (middle panel) or 2.8% glycerol plus 0.5% glucose (lower panel). Aliquots were harvested at timed intervals and Pmk1 was purified by affinity chromatography. Either activated or total Pmk1 were detected by immunoblotting with anti-phospho-p44/42 or anti-HA antibodies, respectively. **B**. Strain MI200 was grown in YES medium plus 7% glucose to early-log phase in the presence of 30 mM NAC and resuspended in the same medium with 3% glycerol. Both activated and total Pmk1 were detected as described above.

We have previously described that glucose exhaustion is one of the multiple physiological insults which activate the Pmk1 MAPK signaling pathway in fission yeast [17]. As shown in Figure 1A, removal of glucose by shifting the cells from a rich medium to a similar medium containing glycerol induced a progressive and clear increase in Pmk1 phosphorylation in control cells, reaching its maximum around 90 min, and slowly decreasing thereafter. This alternative carbon source cannot be assimilated unless a minimal amount of glucose is present, and its initial concentration was selected to prevent differential osmotic changes. Virtually the same pattern of activation was observed when the cells were switched to a growth medium employing both glycerol and ethanol as carbon sources (not shown). Interestingly, transfer of exponentially growing cells from rich glucose medium (7% w/v) to osmotically equilibrated medium with glucose concentrations of either 1% or 0.5% did not elicit a significant increase in Pmk1 phosphorylation (Figure 1A), suggesting that full activation of the MAPK cell integrity pathway in *S*. *pombe* only takes place after complete depletion of this carbon source.

In fission yeast glucose deprivation triggers a moderate endogenous oxidative stress which is followed by the induced expression of genes like *gpx1*^+^ (glutathione peroxidase) and *ctt1*^+^ (cytoplasmic catalase). These products play a critical role in the removal of intracellular hydrogen peroxide arising in the change from fermentative to respiratory metabolism [12]. However, preincubation of cell cultures in the presence of N-acetyl cysteine (NAC), a known scavenger for peroxide radicals, did not affect Pmk1 activation in response to glucose withdrawal (Figure 1B). Identical results were obtained when employing other antioxidants like glutathione or alpha-tocopherol (not shown). Hence, Pmk1 activation in the absence of glucose appears due to the lack of this particular carbon source, and unrelated to endogenous oxidative stress.

### A novel mechanism is responsible for Pmk1 activation in response to glucose deprivation

**Figure 2:**
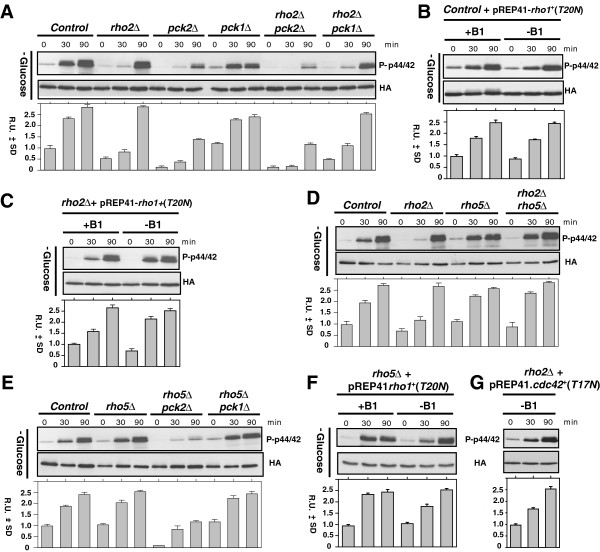
**Glucose deprivation signaling is channelled to the Pmk1 cascade by a Rho-GTPase independent mechanism which involves Pck2. A**. Strains MI200 (Pmk1-Ha6H; Control), MI700 (*rho2Δ*, Pmk1-Ha6H), GB3 (*pck2Δ*, Pmk1-Ha6H), GB35 (*pck1Δ*, Pmk1-Ha6H), GB29 (*rho2Δpck2Δ*, Pmk1-Ha6H), and MM539 (*rho2Δpck1Δ*, Pmk1-Ha6H), were grown in YES medium plus 7% glucose to early-log phase and transferred to the same medium with 3% glycerol. Aliquots were harvested at timed intervals and Pmk1 was purified by affinity chromatography. Either activated or total Pmk1 were detected by immunoblotting with anti-phospho-p44/42 or anti-HA antibodies, respectively. **B**. Strain MI200 (Pmk1-Ha6H; Control) was transformed with plasmid pREP41-*rho1*(T20N), grown in EMM2 medium plus 7% glucose with or without thiamine (B1), and transferred to the same mediums with 3% glycerol. **C**. Strain MI700 (*rho2Δ*, Pmk1-Ha6H) was transformed with plasmid pREP41-*rho1*(T20N). Purification and detection of active or total Pmk1 was performed as described in A. **D**. Strains MI200 (Pmk1-Ha6H; Control), MI700 (*rho2Δ*, Pmk1-Ha6H), JFZ1001 (*rho5Δ*, Pmk1-Ha6H), and JFZ1004 (*rho2Δrho5Δ*, Pmk1-Ha6H), were grown in YES medium plus 7% glucose to early-log phase and transferred to the same medium with 3% glycerol. **E**. Strains MI200 (Pmk1-Ha6H; Control), JFZ1001 (*rho5Δ*, Pmk1-Ha6H), JFZ1002 (*rho5Δpck2Δ*, Pmk1-Ha6H), and JFZ1003 (*rho5Δpck1Δ*, Pmk1-Ha6H), were grown in YES medium plus 7% glucose to early-log phase and transferred to the same medium with 3% glycerol. **F**. Strain JFZ1001 (*rho5Δ*, Pmk1-Ha6H) was transformed with plasmid pREP41-*rho1*(T20N), grown in EMM2 medium plus 7% glucose with or without thiamine (B1), and transferred to the same mediums with 3% glycerol. **G**. Strain MI700 (*rho2Δ*, Pmk1-Ha6H) was transformed with plasmid pREP41-*cdc42(T17N)*, grown in EMM2 medium plus 7% glucose without thiamine, and transferred to the same medium with 3% glycerol.

We next tried to identify the signaling elements involved in the activation of the Pmk1 MAP kinase module in response to glucose exhaustion. Rho2, one of the six Rho GTPases found in *S*. *pombe* proteome, is a main positive regulator upstream of the cell integrity pathway in some stress conditions [18,19]. Importantly, Rho2-dependent regulation of Pmk1 activity is mediated through Pck2, one of the two orthologs of protein kinase C (PKC) present in this organism [8,18,19], while Pck1, the second PKC ortholog, appears to negatively regulate basal MAPK activity by an unknown mechanism [18]. The essential GTPase Rho1 has been also proposed to function as positive regulator of Pmk1 activity [20]. Although we had previously described a partial defect in Pmk1 phosphorylation in *rho2Δ* cells after 90 min in the absence of glucose [18], repeated exhaustive analysis of this mutant under the above conditions showed that maximal MAPK phosphorylation was actually very similar to that of control cells, except for a delay in the activation kinetics at earlier times (Figure 2A). Therefore, this new evidence suggests that the role of Rho2 during signal transduction to the Pmk1 cascade in response to glucose exhaustion is, at most, rather modest.

Notably, MAPK activation was strongly compromised in a mutant lacking Pck2 and slightly affected in Pck1-less cells, whereas simultaneous deletion of *rho2*^+^ in either *pck2Δ* or *pck1Δ* cells did not significantly alter the activation response shown by the single mutants (Figure 2A). These results suggest that Pck2 is the key element involved in full signal transmission of glucose deprivation to the Pmk1 cascade. Moreover, as compared to the Rho2-deleted strain, Pmk1 activation in the absence of glucose remained virtually unaffected in control or *rho2Δ* cells expressing a dominant negative version of Rho1 (*T20N*) (Figures 2B and 2C), which constitutively binds to GDP and behaves like a lack of function version of this GTPase [23,24]. Therefore, neither Rho2 nor Rho1 appear to be major determinants in Pck2-dependend signaling to the Pmk1 MAPK cascade in response to glucose exhaustion.

Rho5 GTPase functions in a redundant fashion to Rho1 and plays a nonessential role during stationary phase and in the process of spore wall formation [25]. It is worth to mention that Rho5 levels are almost undetectable in exponentially growing cells, but increase significantly under glucose starvation [25], thus making this GTPase a potential candidate to modulate Pmk1 activation in a Pck2-dependent fashion. However, as compared to control cells, the enhanced Pmk1 phosphorylation induced by glucose depletion was neither affected by *rho5*^+^ deletion nor modified in *rho5Δ rho2Δ* double mutant cells (Figure 2D). Moreover, simultaneous deletion of *rho5*^+^ did not aggravate the defective Pmk1 activation observed in *pck2Δ* cells (Figure 2E). Notably, Pmk1 activation was still observed in glucose-depleted cells of a *rho5Δ* mutant expressing a dominant negative allele of Rho1 (*T20N*) (Figure 2F). This finding rules out the possibility that both GTPases functionally replace each other during signal transduction to the MAPK module. We also observed a clear Pmk1 activation after glucose exhaustion in *rho2Δ* cells expressing a dominant negative allele of Cdc42 (*T17N*), which is an essential GTPase involved in the regulation of cell morphogenesis in fission yeast (Figure 2G) [26]. As a whole, the above evidences indicate that Rho1, Rho2, Rho5, or Cdc42 GTPases have not significant role in Pmk1 activation in response to glucose limitation, suggesting the existence of unknown additional element/s to activate the cell integrity MAPK cascade via Pck2 under this condition.

### Functional Glucose/cAMP pathway is required for full Pmk1 activation in response to glucose deprivation

**Figure 3:**
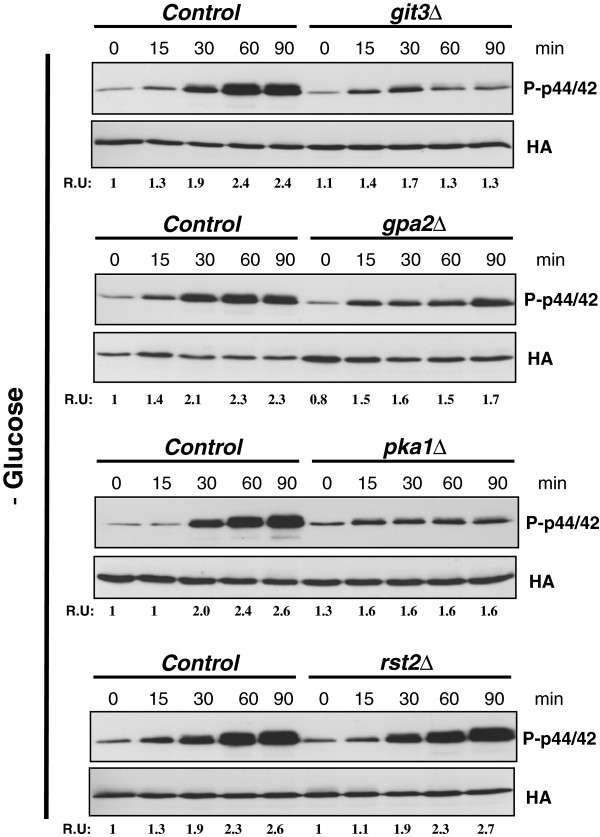
**Functional Glucose/cAMP pathway allows full Pmk1 activation in response to glucose deprivation.** A. Strains MI200 (Pmk1-Ha6H; Control), MM657 (*git3Δ*, Pmk1-Ha6H), MM644 (*gpa2Δ*, Pmk1-Ha6H), MM234 (*pka1Δ*, Pmk1-Ha6H), and MM649 (*rst2Δ*, Pmk1-Ha6H), were grown in YES medium plus 7% glucose to early-log phase and transferred to the same medium with 3% glycerol. Aliquots were harvested at timed intervals and Pmk1 was purified by affinity chromatography. Either activated or total Pmk1 were detected by immunoblotting with anti-phospho-p44/42 or anti-HA antibodies, respectively.

In fission yeast the Glucose/cAMP signaling pathway is involved in the regulation of multiple cellular events, including sexual differentiation, spore germination, osmotic stress response and glucose sensing [14,27]. The main members of this pathway are the G-protein coupled receptor Git3, a heterotrimeric G protein composed of the Gpa2 Gα, the Git5 Gβ, and the Git11 Gγ subunits, plus adenylate cyclase Cyr1, and the cAMP-dependent protein kinase, which in turn is composed by regulatory (Cgs1) and catalytic (Pka1) subunits. In the presence of glucose, Gpa2 Gα subunit binds GTP and activates Cyr1, promoting an increase in cAMP levels which activate Pka1 [27]. Pka1 phosphorylates and negatively regulates the activity of Rst2, a transcription factor responsible for the induced expression of genes like *fbp1*^+^, encoding fructose-1,6-bisphosphatase, whose activity is critical for gluconeogenesis and adaptation to grow on non-fermentable carbon sources (i.e, in the absence of glucose) [14]. Considering such precedents, we analyzed the possible effect of the Glucose/cAMP pathway in Pmk1 activation during glucose deprivation. In comparison to control cells, glucose removal resulted in an important decrease in Pmk1 activation in strains deleted in Git3, Gpa2, or Pka1 (Figure 3). On the contrary, Pmk1 activation remained unaffected in *rst2Δ* cells (Figure 3). These findings suggest that under glucose limitation an operative cAMP pathway is necessary for full activation of the Pmk1 signaling cascade, and that this control is independent on Rst2 function.

### Pmk1 activation in response to glucose deprivation requires *de novo* protein synthesis

**Figure 4:**
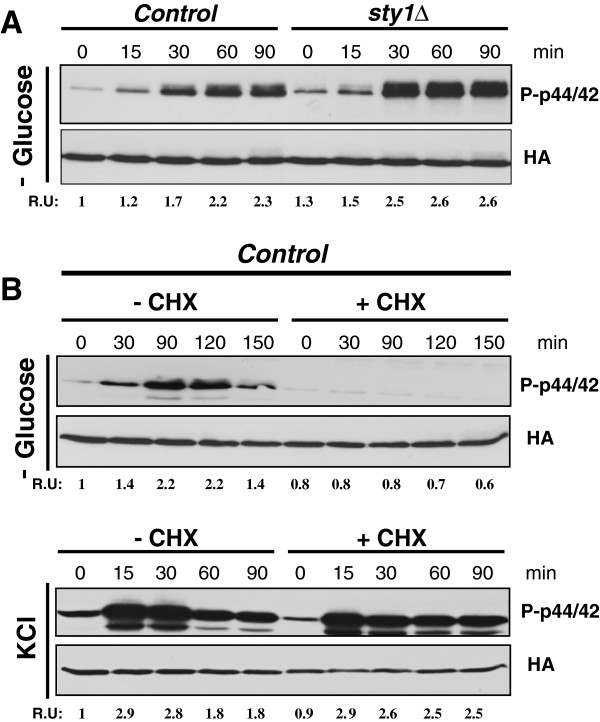
**Pmk1 activation in response to glucose deprivation is independent on the SAPK pathway and requires *de novo *protein synthesis. A**. Strains MI200 (Pmk1-Ha6H; Control), and MI204 (*sty1Δ*, Pmk1-Ha6H), were grown in YES medium plus 7% glucose to early-log phase and transferred to the same medium with 3% glycerol. Aliquots were harvested at timed intervals and Pmk1 was purified by affinity chromatography. Either activated or total Pmk1 were detected by immunoblotting with anti-phospho-p44/42 or anti-HA antibodies, respectively. **B**. Control strain MI200 (Pmk1-Ha6H) was grown in YES medium plus 7% glucose to early-log phase, treated with of 100 μg/ml cycloheximide (CHX) for 60 min, and either transferred to the same medium with 3% glycerol (upper panel) or treated with 0.6 M KCl. Purification and detection of active or total Pmk1 was performed as described above.

To gain further insight into the mechanisms responsible for Pmk1 activation during glucose limitation we analyzed this response in mutant cells of the fission yeast lacking MAPK Sty1, the core element of the SAPK pathway [8]. As shown in Figure 4A, both basal Pmk1 phosphorylation and activation increased in the *sty1Δ* mutant as compared to control cells after glucose withdrawal. This was a result rather expected because of the previous demonstration that Sty1 negatively regulates Pmk1 phosphorylation through the induced expression of several MAPK phosphatases (Pyp1, Pyp2, and/or Ptc1) able to downregulate both Sty1 and Pmk1 *in vivo*[21]. Surprisingly, we observed that pre-treatment of growing cultures of wild type cells with cycloheximide, a protein synthesis inhibitor, fully suppressed Pmk1 activation during glucose exhaustion (Figure 4B, upper panel). Moreover, this response appears to be specific since a strong Pmk1 activation was observed in cycloheximide-treated and untreated cells under saline stress (Figure 4B, lower panel). These results strongly support that in fission yeast the stress by glucose limitation signals to the cell integrity pathway through a hitherto unknown mechanism which requires *de novo* protein synthesis.

### Pmk1 reinforces fission yeast adaptive response to metabolic stress imposed by glucose limitation

**Figure 5:**
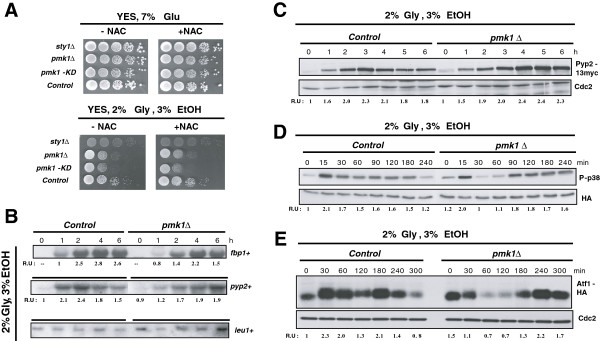
**Pmk1 allows full adaptation to respiratory metabolism in fission yeast by reinforcing the SAPK pathway. A**. Strains MI200 (Pmk1-Ha6H, Control), MI204 (*sty1Δ*, Pmk1-Ha6H), MI102 (*pmk1Δ*), and LS116 (*pmk1Δ*, Pmk1(*K52E*):GFP), were grown in YES medium plus 7% glucose to early-log phase, and 10^5^, 10^4^, 10^3^, 10^2^, or 10 cells were spotted on YES plates supplemented with either 7% glucose or 2% glycerol plus 3% ethanol, in the presence or absence of 30 mM NAC. Plates were incubated for either 3 (glucose plates) or 5 (glycerol plates) days at 28 °C before being photographed. **B**. Strains MI200 (Pmk1-Ha6H, Control), and MI102 (*pmk1Δ*), were grown in YES medium plus 7% glucose to early-log phase and transferred to the same medium with 2% glycerol plus 3% ethanol. Total RNA was extracted, and both *fbp1*+ and *pyp2*+ mRNA levels were detected by Northern blot analysis after hybridization with ^32^P-labelled probes for *fbp1*^+^, *pyp2*^+^, and *leu1*^+^ (loading control) genes. **C**. Strains MI702 (Pyp2-13myc, Control) and LS134 (*pmk1Δ*, Pyp2-13myc), were grown in YES medium plus 7% glucose to early-log phase and transferred to the same medium with 2% glycerol plus 3% ethanol. Pyp2 protein levels were detected with anti-c-myc antibody. Anti-Cdc2 antibody was used as loading control. **D**. Strains JM1521 (Sty1-Ha6H, Control) and MI100 (*pmk1Δ*, Sty1-Ha6H), were grown in YES medium plus 7% glucose to early-log phase and transferred to the same medium with 2% glycerol plus 3% ethanol. Either activated or total Sty1 were detected with anti-phospho-p38 or anti-HA antibodies, respectively. **E**. Strains JM1821 (Atf1-Ha6H, Control) and AF390 (*pmk1Δ*, Atf1-Ha6H), were grown in YES medium plus 7% glucose to early-log phase and transferred to the same medium with 2% glycerol plus 3% ethanol. Atf1 was purified by affinity chromatography and detected with anti-HA antibody. Anti-Cdc2 antibody was used as loading control.

To explore the biological significance of Pmk1 role during glucose deprivation we first determined whether the absence of this MAPK might affect cell viability during growth adaptation from a glucose-based medium to another with a non-fermentable carbon source. In this context, it has been described that the SAPK pathway and its effector Sty1 are critical in fission yeast to allow adaptation from fermentative to respiratory metabolism [12,13]. This is confirmed by results in Figure 5A, indicating that, contrary to wild type cells, the growth of *sty1Δ* cells was impaired when transferred from YES medium to a similar medium in which 7% glucose was substituted by 2% glycerol plus 3% ethanol. The shift to a medium containing 3% glycerol plus 0.05% glucose yielded the same results (not shown). Notably, either *pmk1Δ* cells or a mutant strain expressing a catalytically dead version of the MAPK Pmk1 displayed a growth defect in respiratory medium that was not observed in the presence of glucose (Figure 5A). This defect did not alleviate by the addition of NAC to the culture medium (Figure 5A), suggesting that endogenous oxidative stress was not the cause underlying this phenotype. As a whole, the above evidence supports the idea that Pmk1 activity contributes to cell growth during the adaptation of fission yeast from fermentative to respiratory metabolism.

An attractive possibility about how the cell integrity pathway might favour fission yeast growth during respiration would be that Pmk1 activity positively affects the expression of fructose-1,6-bisphosphatase (*fbp1*^+^), whose activity is critical to achieve growth in the absence of glucose [28]. Confirming this prediction, Northern blot experiments showed that the strong increase in *fbp1*^+^ expression during growth in a non-fermentable carbon source was decreased and delayed in *pmk1Δ* cells as compared to control cells (Figure 5B). Since *fbp1*^+^ transcriptional activation is positively regulated by the Sty1 pathway through Atf1 transcription factor [13], we also analyzed the effect of Pmk1 absence in the levels of Pyp2, a tyrosine phosphatase which dephosphorylates both Sty1 and Pmk1, and whose expression is dependent on the Sty1-Atf1 branch [8,29]. Again, the increased expression of both *pyp2*^+^ m-RNA and Pyp2 protein levels was markedly delayed in *pmk1Δ* cells as compared to control cells after glucose removal (Figures 5B and 5C), thus supporting the conclusion that Pmk1 stimulates the expression of Sty1-Atf1 dependent genes during transition from fermentative to respiratory metabolism.

Because the stress-induced expression of *fbp1*^+^ and *pyp2*^+^ genes is positively regulated by Sty1 via Atf1, we considered the possibility that the delayed expression of both genes in *pmk1Δ* cells during the shift to a non-fermentable carbon source might result from an altered kinetics in the activation of the SAPK pathway. Therefore, we comparatively analyzed Sty1 phosphorylation during glucose deprivation in control versus *pmk1Δ* cells. As shown in Figure 5D, glucose withdrawal induced a quick activation of Sty1 in control cells that was maintained and slowly decreased after 3-4 hours in the presence of non-fermentable carbon sources. However, the kinetics of Sty1 activation in *pmk1Δ* cells was clearly altered, with a more pronounced dephosphorylation after the initial activation, and the activation maintained for longer times (Figure 5D). Similarly, despite a decreased mobility shift and expression observed early after transfer from fermentative to respiratory medium, Atf1 protein levels (expressed as a genomic copy of the *atf1*^+^ gene tagged with two copies of the HA epitope and six histidine residues) remained high in *pmk1Δ* cells at longer incubation times as compared to control cells (Figure 5E). Notably, the late activation of both Sty1 and Atf1 prompted in the absence of Pmk1 is in good agreement with the delayed expression pattern observed for Fbp1 or Pyp2 (Figures 5B and C). Taken together, these results suggest that in fission yeast Pmk1 positively regulates the timely activation of the SAPK pathway during the switch from fermentative to respiratory metabolism.

## Discussion

Several lines of evidence obtained in this work strongly suggest that the signal for glucose exhaustion is channelled to the Pmk1 MAPK module through a mechanism involving unknown elements. While Rho2 GTPase is fully or partially involved in Pmk1 activation in response to most environmental stresses [18], stimulation of the MAPK cascade in response to glucose withdrawal is barely dependent on the activity of this GTPase, since in Rho2-less cells Pmk1 is activated similar to wild type cells except for a slower kinetics at earlier times after carbon source depletion. Lack of function or dominant negative mutants in Rho GTPases like Rho5, whose expression is heavily induced after nutrient deprivation [24], and in Rho1 or Cdc42, which have been mentioned as potential upstream activators of this signaling pathway [17,20], were able to activate Pmk1 in response to this nutritional stress. Moreover, functional screening of Pmk1 activation in mutants deleted in protein kinases involved in nutrient-sensing events (like Tor1, a member of the TOR2C complex [30]; Lsk1, which participates in nitrogen starvation responses [31]; or Ppk9, a Snf1 ortholog required for transcription of glucose-repressed genes in budding yeast [32]) gave negative results as their potential participation in Pmk1 activation (data not shown). Although the identification of the upstream activator/s of the cell integrity pathway during glucose limitation remains so far elusive, our results indicate that Pck2 is a key element for signal reception and transduction to the Pmk1 cascade under these conditions. This conclusion is consistent with the fact that Pck2 is critical for Pmk1 activation in most of the stresses which activate this signaling pathway [18]. However, the detection of some Pmk1 phosphorylation in *pck2Δ* cells suggests that alternative element/s might be able to transduce the activation signal to the MAPK module independently on this particular kinase. Pck1 might be such element, due to the slight defect in MAPK activation observed in *pck1Δ* cells. However, considering that Pck1 negatively regulates both basal and osmostress-induced Pmk1 activity [18], this would imply that it might be playing either a positive or negative role during signal transmission to the cell integrity pathway depending of the nature of the stressing stimulus.

An interesting finding is the observation that *de novo* protein synthesis is necessary to allow Pmk1 activation in response to glucose limitation. Importantly, this appears to be a specific requirement, because translational inhibition did not preclude MAPK activation in response to other stimuli like osmostress. In attempts to find out the identity of inducible element/s we focussed our attention on the SAPK pathway, whose activity is essential in fission yeast to promote cellular adaptation and growth in the absence of glucose [13]. However, mutant strains lacking either MAPK Sty1 or Atf1 transcription factor displayed strong Pmk1 activation in response to glucose withdrawal, suggesting that the SAPK pathway does not perform a significant role in this response. On the other hand, the defective Pmk1 phosphorylation shown in strains deleted in key members of the cAMP pathway gives support to the idea that this signaling cascade contributes positively to Pmk1 activation in conditions of glucose deprivation. However, this interpretation is difficult to understand taking into account that both intracellular cAMP levels and Pka1 activity decrease dramatically with shortage of glucose [27]. Moreover, Pmk1 activation during glucose deprivation was still evident in cells lacking Rst2, a transcription factor whose activity is repressed by glucose via Pka1 [14]. In absence of glucose, lack of Pka1-dependent phosphorylation promotes Rst2 nuclear entry to activate the transcription of a specific set of genes whose products are involved in cellular adaptation to stress (e.g. *ctt1*^+^) and growth in non-fermentable carbon sources (i.e. *fbp1*^+^) [14]. A more likely explanation for the low Pmk1 activation in mutants defective in elements operating upstream Rst2 is that they are constitutively adapted to growth without glucose, and therefore less sensitive to its absence than control or *rst2Δ* cells. However, fission yeast Pka1 becomes hyperphosphorylated during glucose starvation, and it has been proposed that this modification could serve as a mechanism to induce specific PKA functions under limited cAMP-dependent activity [33]. Therefore, the possibility that Pka1 may be involved in Pmk1 activation in the absence of glucose cannot be completely ruled out.

Although the SAPK pathway is critical for growth of fission yeast in the presence of non-fermentable carbon sources, an important demonstration of this work is that full adaptation to respiratory metabolism also requires an operative cell integrity Pmk1 pathway. The functional relationship between Sty and Pmk1 pathways appears to be rather complex. In addition to glucose depletion, several stressing conditions such as hyperosmotic stress, hypergravity, oxidative stress, or thermal upshifts, induce responses involving activation of both Sty1 and Pmk1 [8,17,34], suggesting that the two MAPK cascades show effective cross-talk. As an example, both the basal and the osmostic stress–induced Pmk1 phosphorylation are negatively regulated by the SAPK pathway through Pyp1, Pyp2, and Ptc1 phosphatases [21]. Notably, the fact that the growth defect of cells lacking Pmk1 in the absence of glucose is not as dramatic as in *sty1Δ* cells, suggest that Pmk1 activity may reinforce Sty1 signaling during the control of cell survival and adaptation to these conditions. Results presented here, as the delayed activation of the Sty1-Atf1 branch in *pmk1Δ* cells, the resulting defect in the expression of targets like *fbp1*^+^ or MAPK phosphatase *pyp2*^+^ (and probably others), support this interpretation. Interestingly, Sty1 activation does not become significantly affected in a glucose starved *pck2Δ* mutant as compared to control cells, and Pck2-less cells do not share the growth defect of *pmk1Δ* cells in respiratory media (data not shown). Therefore, contrary to its role as a signaling transducer to Pmk1 cascade in response to glucose exhaustion, Pck2 does not appear to participate in fission yeast growth adaptation from fermentative to respiratory metabolism. It has been described that the transcription factor Atf1 is specifically activated by Pmk1 in response to cell wall stress and regulates gene expression of a limited number of genes [22]. The altered kinetics and defective synthesis displayed by Pmk1-less cells allow to consider that Atf1 is targeted by Pmk1 during glucose limitation in addition to Sty1. However, the altered Sty1 phosphorylation shown by *pmk1Δ* cells also suggests that Pmk1 might regulate signal transduction upstream of Sty1. The identification of specific mechanisms regulating crosstalk between both signaling pathways may deserve further investigations.

## Conclusions

In fission yeast the cell integrity pathway and its key member, MAPK Pmk1, become strongly activated in a transient way after glucose exhaustion. Notably, PKC-ortholog Pck2 is determinant for full activation of this signaling cascade whereas other known upstream elements of the pathway like Rho2 GTPase plays a minor role in this response. These findings, together with the observation that *de novo* protein synthesis is critical for Pmk1 activation, strongly suggest that an unknown branch regulates the signaling of the absence of glucose to the cell integrity pathway. Pmk1 activity is required for fission yeast adaptation from fermentative to respiratory metabolism, as evidenced by the moderate growth defect displayed by Pmk1-less cells in respiratory media. Our results support that Pmk1 reinforces the adaptive response of fission yeast to the nutritional stress by enhancing the activity of the SAPK pathway at two different levels: i- by positively targeting Atf1 transcription factor to allow timely and full expression of genes involved in growth adaptation to respiratory metabolism, and ii- by enhancing signal transmission to Sty1, the core MAPK of the SAPK pathway.

## Methods

### Strains, growth conditions, stress treatments and plasmids

The *S*. *pombe* strains employed in this study are listed in Table 1. They were grown with shaking at 28°C in either YES or EMM2 minimal medium with 7% of glucose (repressing conditions) to a final OD_600_ of 0.5 (actual glucose concentration = 6% as determined by the glucose oxidase method) [12]. Then the cells were recovered by filtration and resuspended in the same medium lacking glucose and osmotically equilibrated with either 3% glycerol, 3% glycerol plus 0.1% glucose, 2.8% glycerol plus 0.5% glucose, 2.5% glycerol plus 1% glucose, or 2% glycerol plus 3% ethanol. In hypertonic stress experiments cultures were supplemented with 0.6 M KCl. In some of the experiments N-acetyl cysteine (NAC; final concentration 30 mM) or cycloheximide (final concentration 100 μg/ml) were added to the glucose-rich based cultures [12]. Plasmids pREP41-*rho1*(*T20N*) and pREP41-GST-*cdc42*(*T17N*) express dominant negative alleles of Rho1 and Cdc42 under the control of the attenuated variant (41X) of the thiamine-repressible promoter *nmt1*, respectively [17]. Cells containing these plasmids were first grown in EMM2 glucose rich medium with or without 10 μM thiamine for about 18 h, and transferred to osmotically equilibrated medium without glucose. Solid media were supplemented with 2% agar (Difco). Transformation of yeast strains was performed by the lithium acetate method [35]. Culture media were supplemented with adenine, leucine, histidine or uracil (100 mg/l, all obtained from Sigma Chemical Co.) depending on the requirements for each particular strain.

**Table 1:** *S*. *pombe *strains used in this study*

**Strain**	**Genotype**	**Source/Reference**
MM1	h^+^	Madrid *et al*. [17]
MM2	h^-^	Madrid *et al*. [17]
MI200	h^+^*pmk1*-*Ha6H*::*ura4*^+^	Madrid *et al*. [12]
MI201	h^-^*pmk1*-*Ha6H*::*ura4*^+^	Madrid *et al*. [12]
LS116	h^+^*pmk1*::*KanR pmk1*(*K52E*)-*GFP*:: *leu1*^+^	Sánchez-Mir *et al*. [36]
MI702	h^-^*pyp2*-*13myc*::*ura4*^+^	Madrid *et al*. [8]
LS134	h^+^*pmk1*::*kanR pyp2*-*13myc*::*ura4*^+^	Sánchez-Mir *et al*. [36]
MI102	h^+^*pmk1*::*kanR*	Madrid *et al*. [8]
TK107	h^-^*sty1*:: *ura4*^+^	Lab collection
MI204	h^+^*sty1*::*ura4*^+^*pmk1*-*Ha6H*::*ura4*^+^	Madrid et al, [12]
MI700	h^+^*rho2*:: *kanR pmk1*-*Ha6H*:: *ura4*^+^	Madrid et al, [12]
GB3	h^+^*pck2*:: *kanR pmk1*-*Ha6H*::*ura4*^+^	Barba *et al*., [11]
GB29	h^+^*rho2*:: *kanR pck2*:: *kanMX6 pmk1*- *Ha6H*:: *ura4*^+^	Barba *et al*., [11]
GB35	h^+^*pck1*::*ura4*^+^*pmk1*- *Ha6H*::*ura4*^+^	Barba *et al*., [11]
MM539	h^+^*rho2*::*kanR pck1*::*ura4*^+^*pmk1*-*Ha6H*:*ura4*^+^	This work
JM1821	h^-^*his7*-*366 atf1*-*Ha6H*:: *ura4*^+^	J.B. Millar
AF390	h^-^*his7*-*366 atf1*-*Ha6H*:: *ura4*^+^*pmk1*::*KanR*	This work
JM1521	h^+^*his7*-*366 sty1*-*Ha6H*:: *ura4*^+^	J.B. Millar
MI100	h^+^*rho5*::*natR pmk1*-*Ha6H*::*ura4*^+^	Madrid *et al*., [12]
JFZ1001	h^+^*rho2*:: *kanR rho5*::*natR pmk1*-*Ha6H*:: *ura4*^+^	This work
JFZ1004	h^+^*rho2*:: *kanR rho5*::*natR pmk1*-*Ha6H*:: *ura4*^+^	This work
JFZ1002	h^+^*rho5*::*natR pck2*:: *kanR pmk1*-*Ha6H*::*ura4*^+^	This work
JFZ1003	h^+^*rho5*::*natR pck1*::*ura4*^+^*pmk1*-*Ha6H*:*ura4*^+^	This work
MM657	h^+^*git3*::*kanR pmk1*-*Ha6H*::*ura4*^+^	This work
MM644	h^+^*gpa2*::*kanR pmk1*-*Ha6H*::*ura4*^+^	This work
MM234	h^+^*pka1*::*kanR pmk1*-*Ha6H*::*ura4*^+^	This work
MM649	h^+^*rst2*::*natR pmk1*-*Ha6H*::*ura4*^+^	This work

*All strains are *ade*- *leu1*-*32 ura4D*-*18*.

### Purification and detection of activated Pmk1 and Sty1

Cells from 30 ml of culture were harvested at different times by centrifugation at 4°C, washed with cold PBS buffer, and the yeast pellets immediately frozen in liquid nitrogen. Cell homogenates were prepared under native conditions employing acid-washed glass beads and lysis buffer (10% glycerol, 50 mM Tris-HCl pH 7.5, 150 mM NaCl, 0.1% Nonidet NP-40, plus specific protease and phosphatase inhibitor, Sigma Chemical). The lysates were cleared by centrifugation at 15000 rpm for 20 min, and the proteins were resolved in 10% SDS-PAGE gels, and transferred to nitrocellulose filters (GE Healthcare). The filters were incubated with either monoclonal mouse anti-Ha (clone 12CA5, Roche Molecular Biochemicals), polyclonal rabbit anti-phospho-p42/44 antibodies (Cell Signaling), or monoclonal mouse anti-phospho-p38 antibodies (Cell Signaling) [12,17]. The immunoreactive bands were revealed with either anti-rabbit or anti-mouse HRP-conjugated secondary antibodies (Sigma Chemical) and the ECL detection kit (GE Healthcare). Quantification of Western blots was performed using Molecular Analyst Software (Bio-Rad).

### Purification and detection of Atf1 and Pyp2

For Atf1 purification (expressed as a Atf1-Ha6H fusion), pelleted cells were lysed into denaturing lysis buffer (6 M Guanidine HCl, 0.1 M sodium phosphate, 50 mM Tris HCl, pH 8.0), and the fusion was isolated by affinity precipitation on Ni^2+^-NTA-agarose beads. The purified protein was resolved in 7% SDS-PAGE gels, transferred to nitrocellulose filters (GE Healthcare), and incubated with a mouse anti-Ha antibody (12CA5). To detect Pyp2 levels (expressed as a Pyp2-13myc fusion) the cleared lysates were prepared under native conditions (see above), resolved in 8% SDS-PAGE gels, transferred to nitrocellulose filters, and incubated with a monoclonal mouse anti-c-myc antibody (clone 9E10, Roche Molecular Biochemicals). Anti-Cdc2 antibody (PSTAIRE; Sigma Chemical) was used as loading control.

### Northern blot analysis

Aliquots of the cultures were recovered at different times, total RNA preparations obtained and resolved through 1.5% agarose-formaldehyde gels, and hybridizations were performed as previously described [35]. The probes employed were a 2.1 Kbp fragment of the *pyp2*^+^ gene amplified by PCR with the 5^′^ oligonucleotide CCGAGAGCGTTTCTTGGA and the 3^′^ oligonucleotide AAGGGCTTGGAAGCCTGG, a 1 Kbp fragment of the *fbp1*^+^ gene amplified with the 5^′^oligonucleotide CTTCCAAGCCAAATACTG and the 3^′^oligonucleotide GATCTCGACGAAATCGAC, and a 1 Kbp fragment of the *leu1*^+^ gene amplified with the 5^′^ oligonucleotide TCGTCGTCTTACCAGGAG and the 3^′^ oligonucleotide CAACAGCCTTAGTAATAT. Ready-To-Go DNA labelling beads and the Rapid-Hyb buffer (GE Healthcare) were used for DNA labeling and hybridization, respectively. mRNA levels were quantified in a Phosphorimager (Molecular Dynamics) and compared with the internal control (*leu1*^+^ mRNA).

### Plate assay of sensitivity for growth

Wild-type and mutant strains of *S*. *pombe* were grown in YES liquid medium (7% glucose) to an OD_600_= 0.6. Appropriate dilutions were spotted per duplicate on YES solid medium supplemented with either 7% glucose or 2% glycerol plus 3% ethanol, and in the presence/absence of 30 mM NAC. Plates were incubated at 28°C for 5 days and then photographed.

### Reproducibility of results

All experiments were repeated at least three times. Depending on the experiment, mean relative units + SD and/or representative results are shown.

## Competing interests

The authors declare that they have no competing interests.

## Authors’ contributions

MM, JFZ, and AF obtained fission yeast mutants. MM and JFZ carried out the experiments to detect activated Pmk1 and Sty1 under different conditions. LSM and TS carried out the Pyp2 and Atf1 detection assays. JVS and JC performed the Northern blot analysis. MG participated in the draft of the manuscript. JC and MM jointly conceived the study and participated in its design, coordination, and draft of the manuscript. All authors read and approved the final manuscript.
